# The ‘invisible homeless’ – challenges faced by families bringing up their children in a remote Australian Aboriginal community

**DOI:** 10.1186/s12889-018-6286-8

**Published:** 2018-12-18

**Authors:** Anne Lowell, Ḻäwurrpa Maypilama, Lyn Fasoli, Yalŋarra Guyula, Abbey Guyula, Megan Yunupiŋu, Jenine Godwin-Thompson, Rosemary Gundjarranbuy, Emily Armstrong, Jane Garrutju, Rose McEldowney

**Affiliations:** 1 Northern Institute, Charles Darwin University, Darwin, Northern Territory 0909 Australia; 2 Yalu Marŋgithinyaraw Indigenous Corporation, Galiwin’ku Community, Elcho Island, Northern Territory 0822 Australia; 3 SNAICC – National Voice for Our Children, PO Box 1445, Fitzroy North, Victoria 3068 Australia; 4 College of Nursing and Midwifery, Charles Darwin University, Darwin, Northern Territory 0909 Australia

**Keywords:** Early child development, Indigenous, Australian Aboriginal, Housing, Crowding

## Abstract

**Background:**

Insufficient and inadequate housing remain serious and enduring problems in remote Aboriginal communities in the Northern Territory (NT) of Australia. Housing is recognised as a key determinant of persisting inequities between Aboriginal and other Australians in health, as well as education and employment outcomes which in turn impact on health. In our qualitative study exploring strengths and challenges related to early childhood in a remote NT community, insufficient housing emerged as the greatest challenge families experience in ‘growing up’ their children.

**Methods:**

The “Growing up children in two worlds” study engaged Yolŋu (Aboriginal) and other researchers in a culturally responsive qualitative research process. Methods included video ethnography and in-depth interviews with six case study families as well as participant observation and interviews with a wide range of other community members. Data collection and analysis occurred through an iterative and collaborative process and the findings related to housing are the focus of this article.

**Results:**

Concerns about crowded and insecure housing were pervasive in the study community where many families are, in effect, homeless. Most rely on extended family to provide accommodation and some never find a secure and stable space in which to bring up their children. Absence of control over their living conditions is a key element underlying many of the sources of distress associated with crowded housing. The lack of food security, sharing sickness and disturbances in the night affecting sleep are just some of the challenges that generate conflict between family members and impact on health, wellbeing, work and school attendance. Although interaction with other family members is highly valued, the ambition of most participants is for independent and secure accommodation in which they can safely ‘grow up’ their children.

**Conclusions:**

Yolŋu who live with the consequences of crowded and insecure housing want their voices to be heard. They best understand the challenges they face and their perspectives must inform the solutions. Equitable access to housing through sufficient and sustained investment in an integrated approach, engaging all stakeholders, is needed. This is essential to address persisting inequities between Aboriginal and non-Aboriginal Australians in health and other outcomes.

**Electronic supplementary material:**

The online version of this article (10.1186/s12889-018-6286-8) contains supplementary material, which is available to authorized users.

## Background

In many remote Aboriginal communities in the Northern Territory (NT), housing stock is insufficient for the population [1, 2] and evidence of associations between housing and children’s health and wellbeing is extensive [3, 4]. Although there has been considerable research related to housing in remote communities, few studies are informed by the perspectives of those who live the experience. In this article we seek to address this gap, sharing the lived experiences related to housing of Yolŋu (Aboriginal) families participating in a broader study of early child development and child rearing in a remote NT community.

There are strong links between housing and child health, educational outcomes and general wellbeing [4–9]. Crowding has been found to contribute to a wide range of health conditions that are highly prevalent in remote NT communities such as otitis media [10] as well as skin and other infections [11, 12]. In these communities rates of acute rheumatic fever and rheumatic heart disease, which mainly result from poverty and overcrowding in childhood, are amongst the highest in the world [11]. In the same community in which this study was conducted, previous research found that young children live constantly with a medium to high-level risk of acquiring infections from environmental contamination [13]. This risk is typically experienced as a result of non-functioning or inappropriate housing hardware and crowding [14]. Initiatives to address such concerns are often developed with little consultation and negotiation with Aboriginal communities and are not long-term or sustainable [14]. Adequate nutrition in childhood also has a critical influence on later health outcomes but can be compromised in crowded conditions [15]. The direct impacts on the health of children are further exacerbated when precarious housing affects the mental health of carers and subsequently their parenting [4].

Remote Aboriginal families are far more likely than other Australians to live in inadequate, crowded, and insecure housing [16–18]. Results of the National Aboriginal and Torres Strait Islander Social Survey (NATSISS) reveal that overcrowding increases with remoteness in Australia, and disproportionately affects the young [19]. In the NT, more than 38% of Aboriginal people live in crowded houses [20] and most (92%) received that classification because they were living in severely crowded dwellings, defined as needing 4 or more extra bedrooms to accommodate the people who usually live there [21].

The level of crowded housing in many remote Aboriginal communities may actually be much more severe than has been reported for a number of reasons. Differences between cultures in the definition of a crowded experience are not reflected in methodological assumptions embedded in the way that census and survey data are collected in Australia [22]. There is also potential to undercount the number of people who are residents as opposed to visitors in a house because many people counted as visitors to communities may be people who are actually homeless [22]. Visitors may have several homes in which they are welcome and between which they alternate for accommodation, none of which is their usual address. This situation could be masking one of homelessness, in which a person desires but cannot obtain a permanent home of their own [23]. Birdsall-Jones, et al. [5] note that crowding may act both as ‘a hedge against primary homelessness and as a force which can impel people into the homeless state’ ([5] p.7) because, while the homeless kin can find shelter in a crowded house, they often create an intolerable situation for themselves and others in the house as it becomes more crowded [5]. As well, crowding statistics can ignore the need for a family to abandon a bedroom because of broken or dysfunctional elements or the use of other parts of the house and yard for sleeping purposes [5].

Sufficient investment in high quality housing could reduce high rates of infectious diseases and improve management of chronic diseases [24] and the need to improve remote housing in the NT has been widely acknowledged for a long time [25]. Through the National Indigenous Reform Agreement under the Council of Australian Governments (COAG), major funding for building new houses and refurbishing those that could be fixed was provided in 2008 to address this need, as part of an integrated approach to ‘Closing the Gap’ on Indigenous disadvantage [26]. This led to the National Partnership Agreement on Remote Indigenous Housing (NPARIH) that aimed to reduce severe overcrowding in remote Indigenous communities through establishment of a 10 year (2008–2018) remote Indigenous housing strategy [26]. However, a review of the NPARIH published in 2017 found that, despite some recent progress, overcrowding rates are still unacceptably high and will become worse without recurrent and additional investment in housing [24].

The consequences of inadequate and insufficient housing are complex and there is a need for better understanding through further research [8, 14, 22, 24]. Although there are numerous studies relating to Aboriginal housing most rely on census or survey data. Qualitative studies that reveal the lived experiences of those who endure the consequences of insufficient housing are largely missing from the literature. In this paper we share the experiences of Aboriginal families in one remote region who live with the consequences of insufficient housing. Their rich descriptive accounts create a comprehensive and nuanced picture of the consequences of insufficient housing for remote Aboriginal people that cannot be captured in statistical data. A greater understanding of such lived experience can inform more adequate and responsive action.

In our study exploring strengths and challenges in early childhood in a remote Yolŋu community of the NT [27], crowding and housing insecurity emerged as the greatest challenges experienced by participating families in ‘growing up’ their children. Yolŋu (Yolngu) are a group of Aboriginal Australians who have inhabited north-eastern Arnhem Land in the Northern Territory of Australia for more than forty thousand years. The study community is located 500 km from the nearest city, and is accessible only by plane or boat. The population is approximately 2200 and 94% are Yolŋu [28]. Yolŋu residents include the traditional custodians of the land on which the community is located as well as Yolŋu from many different clan groups in the region. Connections to traditional lands as well as Yolŋu languages and systems of social organisation and culture remain strong. English is used only in interactions with Balanda (non-Aboriginal people) in limited contexts such as the health centre and the school. The population is steadily increasing, by more than 20% between 2001 and 2011 [29], but this is not matched by an equivalent increase in housing. The study community experienced two severe cyclones in close succession - Lam (February 2015) and Nathan (March 2015) - destroying 80 houses and damaging many others [30]. Many families were relocated to temporary shelters and 3 years later some were still waiting for permanent housing to become available. Crowding continued for many families even after they moved into replacement housing. Although the broader study is ongoing. The consequences of crowded and insecure housing emerged early in the study as a strong theme. Yolŋu researchers and study participants (see further information in Methods) wanted these findings to be shared urgently and widely. Their lived experiences related to housing are the focus of this article.

## Methods

In response to community concerns that Yolŋu knowledge and priorities related to early childhood were not recognised in policy and practice, a qualitative study was initiated to privilege Yolŋu voices in identifying and communicating strengths, as well as the challenges they experience, in ‘growing up’ their children [27]. The “Growing up children in two worlds” study design was informed by extensive previous research in this cultural context [31–33] and incorporated multiple qualitative methods in a collaborative community-based approach. Phase 1 of the study commenced in 2013 to explore strengths and challenges in growing up children from the perspectives of Yolŋu families. The study was then extended in 2016 at the request of participants. Phase 2 (2016–18) continued the research activities commenced in Phase 1 but included a particular focus on strengthening the evidence base for culturally responsive and relevant early childhood assessment processes and support in this cultural context [27]. Longitudinal case studies were conducted with six families over 5 years, combining in-depth interviews with video-reflexive ethnography. Video recordings of participants in their natural environment (ethnography) enabled a ‘reflexive’ process involving participants in exploring the video footage [34]. As well, interviews were conducted with other community members to further explore findings as they emerged from the case studies. The findings related to crowded and insecure housing were derived from data collected from all sources, primarily in the first phase of the project. Further elaboration and confirmation of emerging findings continued during Phase 2. The study did not focus on housing specifically but sought to understand the range of challenges and strengths related to child development and child rearing experienced by Yolŋu families. Crowding and housing insecurity emerged as the greatest challenge for case study families and the consequences of crowding they described were confirmed and elaborated through a process of theoretical sampling [35] during interviews with other community members and feedback sessions with a range of community groups. These rich empirical data [35] provide insights into the lived experiences related to housing of participants over a five-year period from 2013 to 2017.

### The research team

The study was conducted through a partnership between Charles Darwin University (CDU) and the Yalu Marŋgithinyaraw, a community education and research organisation based in the participating community. Collaboration with the Secretariat of National Aboriginal and Islander Child Care (SNAICC), the non-governmental peak body representing the interests of Aboriginal and Torres Strait Islander children, supported wider engagement to explore relevance of findings beyond the study location and to promote translation into policy and practice. The research team included three experienced (authors LM, RG and JG) and three emerging (YG, MY and AG) Yolŋu researchers from the study community as well as four researchers from CDU (AL, LF, EA and RM) and an Aboriginal researcher from SNAICC (JG). The Yolŋu researchers bring valuable ‘insider’ perspectives to the research process and interpretation of findings. They share the languages and cultural background of participants, live in the same environment and experience the same challenges. They bring deep existing connections to all other Yolŋu in the community through kinship and other cultural systems: this enables a level of engagement that outsiders cannot achieve. As well, the cultural and linguistic expertise they bring as core members of the research team is crucial to ensuring ethical and culturally responsive research. Other team members have many years’ collaborative research experience with remote Aboriginal community members in the participating and other remote communities in the NT. Yolŋu team members guided the research process and all were involved in research design, data collection, analysis and interpretation, as well as dissemination of findings.

### Participants

Six children (three boys and three girls ranging in age from 2 months to 2 years at commencement), their younger siblings born during the study period and interested family members participated in longitudinal case studies (see Table 1). Interviews were also conducted with an additional 30 community members (ranging in age from 18 to 70 years) from other clan and family groups. All Yolŋu have established connections to each other through their traditional kinship system and all belong to one of 16 subsections or ‘skin groups’. In reporting the findings, case study children are identified by their ‘skin name’ or similar cultural group name that does not identify individuals as these names are shared by hundreds of other Yolŋu. Other participants are identified through their relationship to a case study child or through another term of their choice.

**Table 1:** Case study participant summary

Mälk (subsection or ‘skin’ name)	Date of birth:	Gender	Bäpurru (Clan/language group)	Family members participating in video analysis and multiple interviews:
Gudjuk	March 2011	Male	Gupapuyŋu	Mother Mother’s sister, Grandmother
Murimuri	June 2011	Female	Ritharrŋu	Mother Mother’s sister, Grandmother
Murimuri’s younger sister	May 2016	Female		
Baŋadi	September 2011	Male	Gumatj	Grandmother, Grandfather
Wamutjan	February 2012	Female	Djambarrpuyŋu	Mother Father Grandmother’s sister Father’s sisters (2)
Gutjan	September 2012	Female	Gumatj	Mother Mother’s sisters (2)
Gamarraŋ	November 2012	Male	Djambarrpuyŋu	Mother Father Father’s sisters (2)
Gamanydjan (Gamarraŋ’s younger sister)	February 2016	Female		

Participation in either case studies or community interviews was entirely voluntary. Some community members asked to participate when they became aware of the study and others were invited to participate by Yolŋu researchers, using purposeful sampling [36] to ensure diversity in terms of age, clan group, socio-cultural and educational background. Participants’ preferred language, which was usually a local Aboriginal language, was used in every stage of the research, to ensure consent for participation was genuinely informed and optimal communication was achieved in data collection, as well as in feedback and confirmation of findings.

### Data collection

Longitudinal case studies were conducted with six families combining data from a range of sources. Children and their families engaging in everyday activities – at home, out hunting, at ceremonies – were video recorded approximately every 3 months from 2013 to 2015 then approximately every 6 months from 2016. Case study family members watched and discussed the videos and these discussions were also recorded. This process elicited detailed interpretations of what was important in child development and child rearing from the perspectives of participants. This is a form of video-reflexive ethnography, a methodology commonly used in health care studies [34], that has also been used in previous research in this cultural context [31, 33]. Numerous further informal discussions with these families occurred when they wished to share additional information and these were also recorded.

Interviews were also conducted with other families and key community informants with a particular interest in early childhood for comparison with data from the case studies. Yolŋu researchers, sometimes with other members of the research team, conducted interviews using a culturally congruent conversational approach [37], exploring participants’ perspectives on what is important in early child development and child rearing (see Additional file 1). Participant observation and interpretation of the video and interview data by Yolŋu researchers provided further insights. Data from all sources were audio or video recorded then translated into English by Yolŋu researchers and transcribed in collaboration with other members of the research team.

### Data analysis

Data collection and analysis occurred simultaneously through a collaborative and iterative process, involving both Yolŋu and other researchers. Initial coding was followed by focused coding, using an inductive process in which codes were derived from the data, and analytical categories were identified through a process of constant comparative analysis [35]. Emerging categories were elaborated, refined and confirmed through further discussions with Yolŋu researchers, participants and other community members. Qualitative data management software, QSR NVivo 11, was used to assist with data organisation.

Illustrative quotes from participant interviews were selected through collaborative discussion within the research team. All quotes were checked again with relevant participants for accuracy and to confirm consent for inclusion in this publication, as well as their preferred form of attribution. Quotes from Yolŋu researchers provide further insights and syntheses of perspectives shared by participants, privileging the voices and interpretation of the cultural group (Yolŋu) rather than the interpretation of researchers who do not share the language and cultural background of the study participants.

Ethical approval was obtained from the Charles Darwin University Human Research Ethics Committee (Phase 1 2013–15 H16025; Phase 2 2016–18: H12136). Approval for the research was also obtained through the relevant Shire Council Local Authority. Senior Yolŋu researchers and the Backbone Committee (the community advisory group for the project) ensured the study was responsive to ethical considerations relevant to the local cultural context.

## Results

**Fig. 1:**
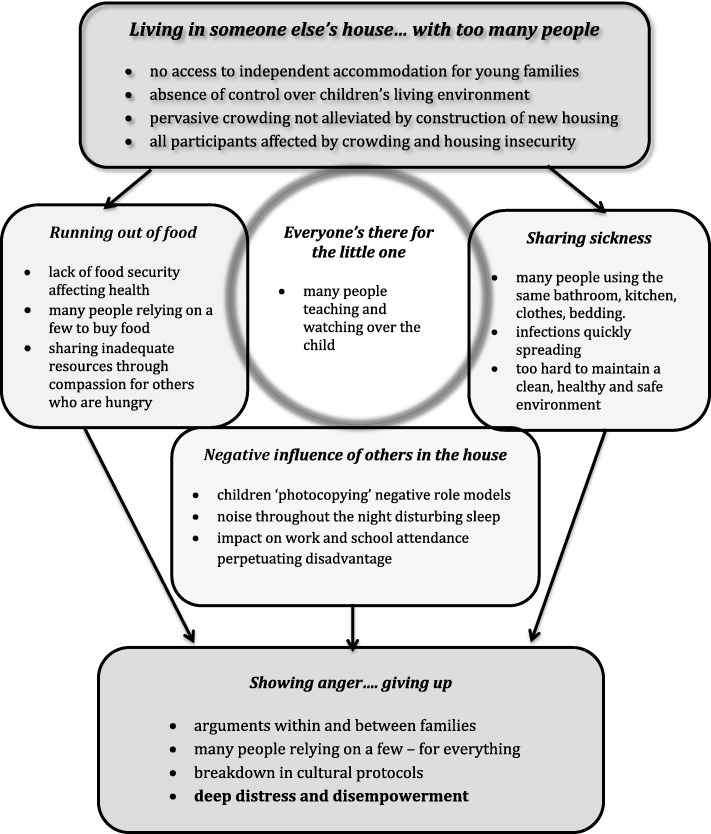
The influence of crowding and housing insecurity on young families

In the study community public housing is the only option. As existing housing stock is insufficient for the population, families often remain on a housing waiting list for years. Most young families have no access to independent housing and therefore rely on extended family to provide accommodation. Some never find a secure and stable space in which to bring up their children and are, in effect, homeless. However, their situation is ‘invisible’ as this form of homelessness is masked by the compassion of others who provide shelter in or outside homes that are often already overcrowded. Six key themes related to housing (see Fig. 1) emerged from an analysis of 30 interviews with community members and multiple in-depth discussions with 15 case study family members. Yolŋu researchers, as insiders sharing the lived experience of participants, brought a deep understanding to data analysis and interpretation. As well, participant observation by Yolŋu researchers and reflections from their own experience were recorded and included in data analysis. Quotes from the Yolŋu researchers are used when they provide further insight, clarification or explanation of experiences shared by community participants.

A central theme - ‘*Living in someone else’s house with too many people*’ – reflects the interlinked concepts of crowding and housing insecurity and illuminates the absence of control over the conditions in which families are bringing up their children. These two elements – housing insecurity and insufficiency - underpin many challenges associated with housing. Although all participants value the support from, and connection with, extended family – ‘*everyone’s there for the little one*’ - almost all long for a place of their own. ‘*Running out of food*’, *‘sharing sickness’* and ‘*the negative influence of others in the house*’ are often overwhelming sources of distress that also generate ‘*conflict and cultural challenge*s’.

### Living in someone else’s house… with too many people

All the participating families - parents and their children - were living together in one room in someone else’s house during the study period, sharing a kitchen and bathroom often with many other people:

*Married and still living with their parents and having their children in that same family, like breathing in one house, every single day, like breathing, breathing, breathing. And no room...* (Yolŋu researcher’s observation).

This comment brings into focus the pressure Yolŋu feel from overcrowded living conditions, obliging families literally to breathe together. Most moved from house to house during the period of the study, some many times, and a few spent time in a tent in someone’s yard. For example, Gudjuk’s family (parents and three children) have been on the public housing waiting list for years but still have no home of their own. During one period, when Gudjuk was four, this family moved between three houses within a few weeks – from a tiny room in the home of his father’s extended family to a new house that was already crowded with 17 occupants, and then to a Homeland (a small community located on land to which residents have a particular cultural connection) a few kilometres from the main community. Living on a Homeland meant relying on inconsistent and expensive generator power. Travelling to school and work was a constant challenge with no transport of their own.

Other families have experienced even more insecure housing: Gamarraŋ’s family has moved many, many times since Gamarraŋ was born 4 years ago – from house to house, community to community and sometimes they had no other option than a tent in someone’s yard. Baŋadi’s mother also lived in a tent while she was pregnant but was fortunate that her parents were finally allocated a house soon after her child was born. This house then became crowded and her parents moved to a tent in the yard. Another mother described her sadness that her child’s parents and grandparents could not live together but were scattered across different, distant communities as they have no access to housing in the community they consider their home.

All of the families talked about wanting their own house where they could bring up their children without the problems of overcrowding. Although most participants described some advantages from living with family members, the challenges from living amongst too many people were overwhelming:

*I want to be away from my relatives. Get a house where there (are) neighbours I don’t know* (Gudjuk’s mother).

Only a generation ago crowding was not such a problem but the community population is rapidly increasing. A Yolŋu researcher in her thirties reflected on the changes her generation have experienced:

*We had our own rooms in those houses … in the old days. And it was quiet and we could read. We had a lot of books to read and we had games, video games. When we had our kids, we had to have them in crowded places…*

In the past, families lived in a house according to their association with a place. Individual families had their own houses. This relationship of a family to a particular part of the community continues but there are more people now and not enough houses.

At any time, young families might have to move for a range of reasons: due to conflict, lack of food security or if other people, who have stronger connections with the leaseholder or even more urgent need, take over their room. This lack of secure housing means such families live with constant uncertainty and all the case study families were forced to move for one or more of these reasons, some many times. Some of the families have moved away from the community for varying periods of time to find accommodation, with different degrees of success, as living in a larger distant town brings many other challenges. Most have applied to Territory Housing (the provider of public housing in the community) but all have waited many years and none of these families have been allocated a house during the study period.

### Everyone’s there for the little one

All participants shared their distress about living in crowded houses where parents have so little control over the environment in which they are bringing up their children. However, some explained that there are also benefits from living with many family members:

*Get together for the little one. So there’s everyone there for the little one, nurturing, sharing responsibilities. And teaching the little one* (Baŋadi’s grandfather).

In a crowded house children can learn from family members of different ages, providing greater support for children’s learning than can be provided by parents alone. Opportunities for children to acquire cultural knowledge that families consider important are enhanced when many people are continuously interacting with the child:

*…Raypirri (strong education about the right way to behave) and also gurruṯu (kinship) and rom (law). Family education…* (Gudjuk’s mother).

This learning is reinforced until the knowledge is internalised by the child:

*It’s like everyone will talk to the child, his mother, his father, his aunty, his uncle, grandmother, grandfather and everyone tells him the same thing… Talking with strong voice to your child, day and night, and your child will listen, keep repeating the strong words and they will hear it over and over and finally it is their idea.* (Yolŋu researcher’s observation).

Many family members living in the house also means that there are more people watching over children to protect them and keep them safe. The support and connection with other family members is central in the lives of all participants but the distress from the negative aspects of living in crowded conditions – sometimes with people who are not close family – was often overwhelming for a range of reasons as further explained below.

### Running out of food

Lack of food security is a frequent problem, affecting the health of both children and adults. Days without food are common. Families often share food when they have the money to do so and then rely on those they have helped to reciprocate later, when they have money. However, even when people have good intentions, this reciprocity is not always possible or is not offered consistently: money may not always be available when it is needed, some people in the house never buy food or people from other houses also come for food.

Parents struggle to prevent their children from being underweight. For example, one of the study children became underweight because of her living conditions in a crowded house:

*the mother wasn’t getting food because of a crowded house and food runs out and it was too hard to feed herself for her breastfeeding. And also the child (wasn’t getting food)* (Yolŋu researcher recounting her observations of Wamutjan and her mother).

Another mother talked about her struggle to feed her children in her current living conditions and her dream of not having this worry:

*The reason that my kids are not growing is because I need a house. To feed the kids properly... I’m in the city, I’ve got my own house. I send them to school. That’s what I’m dreaming* (Gudjuk’s mother).

Some families will try to keep food in their rooms, but this can be difficult when other people in the family are hungry:

*We buy food and everyone relies on one Yolŋu and then the food is all gone… sometimes, no food - 3 days, 4 days. We saved food for Gamarraŋ and mother and father had no food, only water. If we have our own house we will have plenty of food in the house. We are finding it too hard* (Gamarraŋ’s mother).

Some people will spend their money on things other than food, for example gambling, and then rely on others in the house to buy the food and pay for power. Overcrowding forces many people to rely on take-away food, even though this is an expensive and often unhealthy option, because they cannot keep food in their house:

*… and then, you know, you get paid and then you think ‘Ah, I’m not going to buy food for everybody!’ and then the fridge ends up empty, and cupboard, and then ‘I might as well just go up to the take away and have my feed there’ and then pretend I’ve got no money - so I can’t afford to feed anybody … there’s just too many people...* (Baŋadi’s grandmother).

One of the Yolŋu researchers described the conflicted feelings she has about sharing food, an experience shared by many of the families:

*Now I’ve got no money for food, I’ve got no food in the house… ‘cause everybody keeps coming and getting from us. And it’s really hard, I try hard to hide things from people but it’s wrong to do that… I just give it away… Because I feel that person’s really sick and hungry. But another time, I get sick and hungry*.

As this example illustrates, in the midst of their own hardship these families feel compassion for others, so they help other people in the house when they are hungry and then their own food is gone.

### Sharing sickness…

Yolŋu are aware of the way that sickness is spread due to too many people in a small space sharing everything. This is a constant worry for many families, as one of the Yolŋu researchers explained, based on her own experience and observations of other families:

*We share scabies. We share sores. Flu… One person gets to another person, to another person. But it still hangs around at that house. Because why? A lot of people in that house… like one person has to do the cleaning up, then, 5, 10 min - you see the house gets messy again. Yolŋu houses never stay clean because why? Many people in the house. That’s why it’s…. really hard to make it (a) clean, healthy environment, safe environment*.

In most houses, while many people share the same toilet and shower, only a few take responsibility for cleaning. Bedding, plates, cutlery and cooking utensils are in short supply when many people borrow items and don’t return them so everyone has to share the few things available:

*…a lot of people get flu and it spreads to the kids… sharing drinks, sharing food, sharing one spoon… a lot people using one toilet and one shower...* (Gamarraŋ’s parents).

*To live in one house and share everything… so sometimes some things (sheets, blankets, pillows) that get used, it rotates around three bedrooms. I don’t like that. That’s why I want to have my own house* (Gudjuk’s mother).

Bilinydjan, a mother of two, talked about how often her own children were sick when she lived in a crowded house with eight other children, sharing everything with many other people:

*Same kitchen, same bathroom, same toilet, same dishes, everything, same.*.. (Bilinydjan, community member).

Many people will also share the same washing machine, not only those that live in the house but also other family members and neighbours who don’t have one of their own. A functioning washing machine can soon become overwhelmed:

*One bathroom, one toilet, one fridge. I bought new washing machine last year and it only took 3 months, 4 months then (broke)... and sometimes I get worried about the kids clothing. I spend lot of money on the kids clothing. Then the other kids for aunty, sister, brother in law’s kids, they’re using my kids’ clothes* (Gudjuk’s mother).

Children’s safety and the potential for accidents created by many people in the house is another concern:

*Yeah... overcrowding and, you know, people can just go, cut up a fish and leave the knife where it’s really easy for a kid to reach up and grab it. Anything, even a screwdriver* (Baŋadi’s grandmother).

All the participants recognised that keeping the house, clothing, cooking equipment and people clean is fundamental to good health but found it a continuous challenge in a crowded house. Infrastructure designed for use by one family quickly becomes dysfunctional when shared by too many people. When one person is relied on to do the cleaning, a common situation, they sometimes just give up in frustration:

*There’s just too many people ... who’s going to do the cleaning, inside and out, and then it’s left up to one person to do the work and they don’t realise, you know, how hard it is for that person, ‘cause there only certain people that… have eyes to see all the mess around…We’ve had a lot of times when we’ve argued about who’s going to be keeping the house clean, can’t be just one, two people. But I think… it’s probably … ‘It’s not my house. I’ve got my room so I look after my room, not going to bother about the dishes. You know, I didn’t make that mess!’* (Baŋadi’s grandmother).

This absence of shared responsibility for shared spaces is inevitable when people living in a crowded house that is not their own do not have any sense of ownership or control over their environment.

### Negative influence of others in the house

Participating families often expressed distress about what their children were learning from others living in or visiting their house, a situation over which they have no control. Older children swearing, teasing, playing day and night and no opportunity for young families to spend time together with their children in their own space were common concerns shared by participants. The influence of other children and adults in a crowded house makes it hard for parents to manage their own children. They talk of their children ‘photocopying’ or following the lead of other, usually older children. These concerns were summarised by a Yolŋu researcher:

*…a lot of overcrowding and learning all kinds of different things (different behaviours and attitudes), people using different ways – kids are photocopying, what sort of role model is in the house… it’s the language – picking up the bad language…*

Although there are positive influences on learning from having many people in the house, as described above, this is not always the case. School attendance, which is consistently low in the study and other remote communities, is also affected by crowded housing:

*Because there is lot of relatives living in one house, sharing one house, like lot of influences from other kids towards my kids and also not enough food for the family… sometimes I give them bath, breakfast, feed them then after bath let them catch the bus to school. But the other kids are staying in the house, if their parents don’t get them to school, my kids see that and don’t want to go… because influence of other kids, visitors and family… that’s why, need to live in my own house. I need a house so I can manage everything for myself* (Gudjuk’s mother).

Many people – children and adults – are active during the cooler hours of the night and those who are not working or at school catch up on sleep during the heat of the day. Because of the noise and distraction from others in the house, crowding makes it difficult for anyone *‘ working, or studying, or doing something really serious’* (Yolŋu researcher’s observation). The difficulty in getting enough sleep – or enough food – in a crowded house affects children’s school attendance and their ability to concentrate when they do attend. These challenges also impact on the ability of adults to work which further impacts on children’s health and wellbeing when their parents are unable to earn an income.

Children living in a crowded house also struggle to find a quiet place to do homework and to keep their books safe, further compromising the opportunity for children to succeed at school. A place of their own where they can bring up their children is the dream of many families, as this example from Gamarraŋ’s father illustrates:

*(If we have our own house) the child can think the right way - (from too many kids) they learn the wrong way - not their own thinking … if they can follow their own way they would forget the influence of other kids. I will try to teach them a good way - listening, responsible… What kind of child would that child turn out to be, living like this? That’s why I want to be away from family. I try hard to move away from family…* (Gamarraŋ’s father).

These consequences of crowded housing impact on children’s health, behaviour, school attendance and educational achievement, as well as adults’ participation in employment. Crowding undermines the aspirations families have for their children and exacerbates their sense of powerlessness and frustration when they have no access to the solution – a home of their own.

### Showing anger… and giving up

Conflict amongst families is common in crowded living conditions where arguments over roles and responsibilities as well as access to scant resources can lead to stress, as the examples above have illustrated. In addition, when problems occur between children, this can lead to conflict between their parents and other family members, for example:

*Sometimes (we argue) when the kids…tease each other (my kids and my brother’s kids)* (Murimuri’s mother).

Arguments within or between families may compel one family to shift to other accommodation if an alternative is available. However, there is often no other option forcing Yolŋu to remain in stressful situations with little or no possibility of removing themselves or their children away from conflict.

Crowded housing also undermines important cultural protocols, for example the avoidance rules between mother-in-law and son-in-law and between brothers and sisters, as described by one of the Yolŋu researchers:

*when a woman is pregnant she keeps away from her brothers...also covers herself with a towel or sarong – in the past she would have moved away from her mother and brothers to go with the husband so (it) wasn’t a problem…*

There are many restrictions on speech and behaviour in the presence of men who are in the kinship category of ‘brother’, not just brothers who share with their sister a biological parent. Complying with these protocols can be extremely challenging when sharing the same restricted living space. Without the option of moving away, further stress and conflict result when cultural protocols are compromised.

Managing very limited financial resources was also a challenge and another common source of conflict. Although most participants were living on government benefits, even those with jobs would spend days between payments with no money at all. Food is expensive in the study community and any income was quickly consumed buying essentials and sharing with family. Money and housing are just two of the many profound, and relatively recent, changes from colonisation impacting on Yolŋu lives.

The challenges of crowded and insecure housing – not enough sleep, not enough food or energy for school or work, the negative influence of others in the house, sharing sickness, conflict and stress – were shared by all participants. Many were frustrated that the reality they live with every day is not recognised beyond the community and they are blamed for problems that they have no power to prevent. All were striving to provide a supportive environment in which to bring up their children, but disempowerment and defeat were often the result of these constant challenges:

*… they just give up. They’ve had enough. They lose their courage* (Baŋadi’s grandmother).

## Discussion

The impact of insufficient and insecure housing on the families’ capacity to ensure the health and wellbeing of their children was a source of deep and pervasive distress for the participants in this study. Insufficient housing forces reliance on others for accommodation. Such insecurity of tenure means young families have little control over where they live or the environment in which they are bringing up their children. When people are living in such crowded conditions and do not have control of, or access to, space for social relations, they are considered homeless [38].

In 2007, Wild and Anderson described the shortage of housing for Aboriginal people in remote communities as ‘nothing short of disastrous and desperate’ ([39] p.197). Despite ongoing housing renewal programs, our findings and those of other studies in the region [13, 40] illustrate that the rate of increase in housing supply does not meet the rate of increasing need. This means that construction of new housing does not necessarily result in reduced levels of crowding [1] and, therefore, there is no significant improvement in the wellbeing and happiness of tenants [41].

All of the participating families moved between houses – some many times *–* during the period of our study. Their options were limited to a single room in someone else’s house or sometimes a tent outside when others had priority for the rooms inside. According to the national census data [16, 28] average household occupancy in the study community has barely changed between 2011 (average of 5.3) and 2016 (5.4) and remains double the national average [28]. The accounts of our participants support previous research in the same community that found the mean number of persons per bedroom was 3.4 in houses where young children were living [13] revealing that the actual level of crowding is much more severe than census figures would indicate. Such discrepancies between official statistics and the reality experienced by Aboriginal people have also been found in other contexts [42].

The influence of overcrowding on health and social and emotional wellbeing remains a key concern for Aboriginal people in remote communities [40] as well as in urban contexts. For example, the findings of a recent study [42] in urban Sydney were strikingly similar to those of our study. They found housing issues to be a pervasive source of stress in people’s lives due to: insufficient access to space, privacy and basic amenities; feeling powerless to change their housing situation; and stress on relationships affecting individual emotional wellbeing [42]. The potential protective influence of extended family for the development of children growing up in crowded households has been suggested [7, 9]. The findings of our study regarding the rich and intensive learning environment in which Yolŋu children grow up (to be reported in a subsequent publication) provide some support for this suggestion. However, Yolŋu families also experience deep distress due to housing insecurity and insufficiency that they strongly believe has a negative impact on the health and wellbeing of their children.

Lack of control in maintaining an adequate and consistent supply of food was a recurring theme in our study, resulting in families experiencing days without food and difficulty in maintaining a healthy weight for children. Even a short period of low money may contribute to reduced diet quality and exacerbate cardio-metabolic disease risk [43, 44]. Sharing is considered an integral value of Aboriginal identity [14, 45], as our findings clearly illustrate, and compassion for other family members, particularly children, meant that food was shared even when there was no money to buy more. The consequences of such sharing, often unreciprocated, resulted in extended and frequent periods of hunger for many participants and their children.

The worry expressed by participants about sickness spreading due to too many people in a small space, sharing one bathroom and kitchen, is well justified as previous research demonstrates e.g. [10, 11]. Disrupted and inadequate sleep was another important source of distress for the families in our study and for other families across the region and beyond [14, 40] affecting children’s attendance and performance at school as well as adults’ participation in work, perpetuating economic disadvantage. Ensuring sufficient sleep is essential for both physical and mental health but is difficult to achieve in a crowded house. A recent study in Western Australia found that children’s sleep duration decreases with increased numbers of adults in the household, with important implications for future health outcomes for Aboriginal children, including metabolic dysfunction and other chronic illnesses [43]. The contribution of insufficient sleep, as experienced by participants in our study, to the high rates of chronic health conditions in their region is yet to be explored.

Study participants also identified the negative impacts that their crowded and insecure housing has on their ability as parents to manage their children’s behaviour. The influence of other, often older, children and no opportunity for young families to spend time together and with their children in their own space, were common concerns.

As noted by other researchers [3, 41, 46, 47], in combination with other factors, there is a strong relationship between remote NT Aboriginal children’s poor attendance at school and their crowded living conditions / homelessness. Disruption from changing households has also been found to have a negative effect on participation in early childhood education [48].

A recent study revealed that having fewer people in a new or refurbished house was a key factor in improving Aboriginal tenants’ living conditions because with fewer people in the house, it was ‘easier to work or look for work and school attendance for their children…’ ([41] p.63). These authors also noted that having fewer children in the house was an improvement because it meant that their own children were ‘less likely to be persuaded by other kids not to go to school’ and both children and adults had a better chance of getting a good night’s sleep ([41] p.67). They found, as did our study, when people simply moved from the old to the new house with no reduction in numbers, these improvements were not noted. Therefore, the condition of the house was less important than the crowding in the house.

Houses in the study community have become extremely crowded in large part because population growth has not been matched by housing growth. However, another important factor contributing to crowding is Yolŋu householders’ deeply felt responsibility to provide shelter for kin when they are visiting or are homeless. This finding is echoed in case studies of Aboriginal households in two metropolitan and two regional centres [45]. They found the same commitment to ‘mutual care of extended family’ which they identified as ‘part of the deep structure of Aboriginal culture’ [45] p.3 calling it a ‘cultural driver’ [45] p.2 of household crowding. As such, the crowding can be seen as part of maintaining cultural protocols in a changing world where the living conditions people find themselves in do not accommodate such practices. The importance of kinship connections and associated sociocultural responsibilities can have both positive and negative effects [14, 49]. The lack of privacy in a crowded house often causes a break down in cultural practices such as avoidance protocols, creating serious conflicts and stress for families. Fantin’s study [23], also with Yolŋu, identified these specific avoidance rules as requiring the ‘strictest behaviour patterns’ [23] p.73.

For the Yolngu, avoidance behaviour is best described as a form of strictly choreographed, ritualised respect between parties that manifests itself in people avoiding each other through their physical orientation, spatial distance, and visual and verbal behaviour…Violation of such prescribed behaviour patterns may cause shame, fear and anger, and sometimes lead to violence [23] p.73.

The lack of control felt by participants in being able to manage these cultural as well as other issues was often a source of distress and cause of conflict, a point also made by Memmott et al.:

Loss of control over who stays in the house created a sense of crowding and stress for householders. Loss of privacy and the ability to choose who to be with at any particular time was a significant source of stress [45] p.3.

Loss of control may actually be a more significant trigger of stress than the number of people staying in the house [45]. Absence of control was central to the experiences of participants in our study who expressed a strong desire for greater autonomy, and therefore control, in protecting their children from sickness, over their food supply, infrastructure, the influence of others on their children, noise levels and visitors. Conflict within and between families is a frequent and distressing consequence of housing instability and overcrowding in the study community as well as other contexts [14, 24, 40].

The pervasive and damaging influences of insecure and crowded housing on health and wellbeing, education, employment and cultural strength are clearly illustrated by the findings of our study. Current levels of government investment in public housing in remote communities will not meet the increasing levels of need [24]. An integrated approach to addressing this need has repeatedly been suggested [4, 40, 50], acknowledging the interconnections between health, housing and other social determinants such as education and employment. A ‘whole of government, whole of community approach to decision making’ ([50] p. 15) has been strongly recommended by both government and remote community members [50]. Sufficient investment in appropriate housing to meet current and future needs is essential but sustainable and effective solutions can only be achieved when Aboriginal people are genuinely engaged in decisions. Those most affected – the ‘invisible homeless’ such as the participants in our study – also want their voices to be heard.

This study has focused on one remote area to enable in-depth exploration of the topic from the perspectives of one cultural group. Therefore, it cannot be assumed that the findings are relevant to other cultural groups or locations. However, our findings are consistent with the few previous related studies in the same and other Aboriginal communities in Australia, both remote and urban.

## Conclusions

The lived experiences of the families participating in this study reveal the challenges and distress Yolŋu experience from living and raising their children in crowded and insecure housing: days without food, disturbances throughout the night, conflict and negative influences from others on their children’s behaviour, little if any control over the conditions that they know will influence the health and wellbeing of their children. These findings challenge the common assumption that Yolŋu live with many family members by choice. Deep and valued relationships with other family members are crucially important but most participants in this study dream of achieving a level of autonomy and independence that allows control over how, and with whom, they ‘grow up‘their children.

The impacts of crowding and housing insecurity on health as well as social and emotional wellbeing, education and employment, which in turn impact on health, are clearly demonstrated by our findings and other studies. Continued failure to adequately address housing insufficiency for this and future generations of Aboriginal children and their families perpetuates the serious and pervasive disadvantage they experience. Co-ordinated action, engaging all stakeholders, to achieve equitable access to housing in remote communities, can reduce persisting inequities between Aboriginal and non-Aboriginal Australians in health and other outcomes. Such action must be informed by the people who know best what is needed – Aboriginal families who currently live with little hope of escaping the consequences of crowded and insecure housing.

## Additional file

Additional file 1: Interview Guide. (DOCX 14 kb)

## Abbreviations

Aboriginal and Torres Strait Islander peoples: This term is used when referring to populations across Australia, acknowledging the diversity of cultural groups to which the term refers through use of the plural form when appropriate
Aboriginal: This term is used when referring to regions that include cultural groups other than Yolŋu but do not include Torres Strait Islander communities
Balanda: Is the term used by Yolŋu to refer to non-Aboriginal people
Indigenous: This term is only used when required for consistency with specific publications referred to in the text when they use the term ‘Indigenous’ to refer to Aboriginal and Torres Strait Islander peoples of Australia
NT: Northern Territory
SNAICC: Secretariat of National Aboriginal and Islander Child Care
Yolŋu: This term refers specifically to Aboriginal people of North East Arnhem Land, Australia
