# Supplementary material for: The ‘invisible homeless’ – challenges faced by families bringing up their children in a remote Australian Aboriginal community
Source: BMC Public Health. 2018 Dec 18;18:1382. doi: 10.1186/s12889-018-6286-8 (PMC6299657; doi:10.1186/s12889-018-6286-8)
Supplement: Supplementary file 1 — Interview Guide. (DOCX 14 kb) [file 12889_2018_6286_MOESM1_ESM.docx]

**“Growing up children in two worlds” Community and Case Study Interview Guide**

Interviews used a narrative approach to explore participants perspectives about what is important in child development and child rearing in their preferred language using a conversational style that is consistent with local communication protocols. Topics to elicit further discussion (if needed) included:

- What is important in growing up Yolŋu children?
- From when a child is born who is helping them to learn and how?
- What is your child learning now (if relevant)?
- Experience with health issues and nutrition
- Experiences with playgroup / preschool / school
- Ideas about differences in development
- What makes ‘growing up children’ easy and what makes it difficult?

Further discussion points were included to clarify / expand on emerging findings as the study progressed, for example:

- Learning about connections
- Communicating from conception
- Experiences with housing
- Ways of monitoring how a child is learning
- Role of men and boys in growing up children
